# Clinical profile and associated comorbidities of cerebral palsy in children visiting Orotta National Referral Hospital, Eritrea: a cross-sectional study

**DOI:** 10.1186/s12887-024-04938-1

**Published:** 2024-07-18

**Authors:** Yohannes Kibrom, Emnet Tekeste, Sirak Tesfamariam, Zemichael Ogbe, Mahmud Mohammed

**Affiliations:** 1 Orotta National Referral and Teaching Hospital, Asmara, Eritrea; 2 Barentu Military Hospital, Barentu, Eritrea; 3 Product Evaluation and Registration Unit, National Medicines and Food Administration, Asmara, Eritrea; 4 Orotta School of Medicine and Health Sciences, Asmara, Eritrea

**Keywords:** Cerebral palsy, Children, Cerebral palsy subtypes, Comorbidities, Motor function, Eritrea

## Abstract

**Background:**

Cerebral Palsy (CP) is one of the most common physical disabilities in children. This study aimed to explore the clinical spectrum of CP at Orotta National Referral and Teaching Hospital, including CP subtypes, gross motor function, patterns of associated comorbidities, and possible risk factors in children aged 2 to 12 years.

**Methods:**

A hospital-based cross-sectional study was conducted from January to April 2022 in 153 children with suspected motor symptoms. The Surveillance of CP in Europe (SCPE) decision tree was used as an inclusion criteria guideline and the evaluation of the participants was done using a standardized questionnaire and clinical examination. Descriptive statistics, chi-square test, and logistic regression were employed to statistically analyze the data.

**Results:**

Eighty-four children who fulfilled the clinical criteria were included in the study. The median age was 5-years [IQR: 3.8] with an equal distribution of males and females. Quadriplegic CP was the most common subtype (51.2%) followed by unilateral (hemiplegic) CP (22.6%), and dyskinetic CP (14.3%). Most children had severe gross motor impairment GMFCS level IV-V and females were almost three times more likely to have GMFCS level IV/V than males (AOR: 2.70; CI: 1.08–6.72; *p*-value = 0.033.) More than half (52.4%) of the neonates either did not cry within five minutes and/or needed breathing resuscitation, 55.3% had to be admitted to the NICU with a median of 5 days’ hospital stay. Between the first week of birth and the first year of life, 28.6% had trouble feeding, 26.2% had an infection, 10.7% had difficulty breathing, 20.2% had seizures and 6% had jaundice. Feeding problems (64.3%), speech problems of some sort (91.7%), and epilepsy (46.4%) were the most commonly associated comorbidities with CP.

**Conclusions:**

The clinical profile of the CP patients was found to be dominated by the spastic subtype and moderate to severe disability. Since perinatal risk factors were found to be dominant, strengthening maternal and child healthcare systems is recommended to minimize incidents of preventable risk factors and the burden of the disability.

**Supplementary Information:**

The online version contains supplementary material available at 10.1186/s12887-024-04938-1.

## Background

Cerebral palsy (CP) is one of the most common causes of physical and mental disability in children worldwide [1–3]. CP refers to a heterogeneous group of conditions involving permanent non-progressive central motor dysfunction that affects muscle tone, posture, and movement. These conditions are due to abnormalities in the developing fetal or infantile brain resulting from a variety of causes [1, 4–7]. The motor disorders of CP are often accompanied by problems with cognition, communication and behavior, epilepsy, secondary musculoskeletal problems and others [8].

Even though the overall global prevalence of CP is not exactly known due to a lack of standardized data registries and research across all continents, it is estimated to be around 2.0 to 2.5 per 1000 live births. Despite the advancement of medical care for preterm and low birth weight children in recent years, the prevalence of CP has remained constant [6, 9–12]. A recent systematic analysis study, however, reports that the prevalence of CP has decreased to 1.6 per 1000 live births in high-income countries (HIC) [13]. Given the paucity of population-based studies in low- and middle-income countries (LMICs), mainly Africa, the exact burden of CP is not known in the underprivileged population [2, 3, 5]. A few rigorous population-based studies recently published in Uganda and Bangladesh revealed that the prevalence of CP in LMICs is more than 2.5 times higher than in HIC [5, 14, 15]. Most of the studies done in LMICs are hospital-based clinical samples and suggest a prevalence ranging from 2 to 10 cases per 1000 children [3, 8].

Generally, the cause of CP is not clearly known, but several risk factors have been associated with it. In a study done by the Collaborative Perinatal Project, in 80% of cases, factors occurring in the antenatal period were identified to cause abnormal brain development, while fewer than 10% of children with CP had evidence of intrapartum asphyxia in developed countries [7]. That being said, however, the causes of CP in developing countries are thought to be different from those in the developed ones [1, 16]. Several studies performed in the LMICs show that perinatal causes, mainly birth asphyxia, tend to dominate, constituting greater than a third of the overall causes followed by post-natal and antenatal factors [17, 18]. These studies clearly show that information from studies conducted in HIC cannot be generalized to the LMIC, and that more studies on CP need to be carried out in LMICs.

There is a significant lack of literature regarding the prevalence, clinical, and sociodemographic features of children with CP in Eritrea. Therefore, this study was aimed to identify the clinical subtypes and motor function, common possible risk factors, and associated comorbidities of CP in the context of Eritrea, a low-income East African country.

## Methods

### Study design and area

This study was a hospital-based cross-sectional study conducted between January and April 2022 in the physiotherapy center of the Orotta National Referral and Teaching Hospital, whose catchment area encompasses the entire country, Eritrea.

### Study population

Children with different disabilities, predominantly motor problems, come to the physiotherapy center to receive different rehabilitative treatments, such as exercise therapy and acupuncture. This population was selected because of the highest number of children with CP per given time compared to the neurology follow-up clinic in Orotta Pediatric Referral and Teaching Hospital or the National Association of Intellectual/Behavioral and Developmental Disability Eritrea. These children are divided into eight groups, and each group comes to the physiotherapy center for a session of two weeks (ten working days) for exercise therapy and acupuncture. Every child between the ages of two and twelve years who was undergoing rehabilitation as a case of CP, including new referrals during the study period, was assessed to be included in the study. Similar to the study by Kakooza-Mwesige et al. [14], the age range of 2–12 was chosen as CP is difficult to differentiate from other disease entities in the early ages [7, 19] and cases above 12-years are subjected to recall bias.

Participants who failed to fulfil both the WHO Screen Questions (Questions 1 and 5) [20] and the ‘Decision Tree’ in the Surveillance of Cerebral Palsy in Europe (SCPE) [19], along with those participants whose caregivers could not provide a full history, were excluded from the study.

### Sample size determination and technique

All patients attending the physiotherapy centre of the Orotta National Referral and Teaching Hospital, including new referrals during the study period were eligible for recruitment.

### Outcome definition measurement

CP: Patients who fulfil the diagnostic criteria of the WHO and SCPE [19, 20].

Epilepsy is defined as a condition of unprovoked recurrent seizures. The classification of epilepsy in this study was based on seizure description alone, according to the International Classification of Epileptic Seizures of 1981 [21].

Speech and language impairments were explained as an inability to use specific words, as well as their comprehension of the child’s maternal language.

Perinatal period: The duration from the 28th week of gestation to the first seven days after birth was considered a perinatal period [14, 22].

### Data collection tool and approach

A structured, pretested, and pre-coded questionnaire (Additional file 1) was adopted upon review of similar questionnaires [14, 19] and was used to interview the caregivers on socio-demographic data, and history of the pregnancy, birth, and post neonatal incidents. The interview was conducted by medical doctors appropriately trained on how to use the questionnaire. Prenatal risk factors included maternal alcohol consumption, smoking, fever/infection, malaria, or abnormal bleeding during pregnancy. Perinatal factors comprised of information on the place of delivery, complications during delivery, prematurity, fetal presentation, and other notable events. As the APGAR score nor the arterial blood gas analysis of all the study participants could be attained, mothers were questioned on how fast their child cried at birth, and the requirement for respiratory resuscitation as a proxy indicator of perinatal asphyxia. Furthermore, potential post neonatal incidents leading to CP were sought and information on co-morbidities was investigated. This included epilepsy, feeding problems, skin lesions related to pressure sores, and speech and language impairment. School attendance rate and performance of the children with CP were asked as well.

Similar to other studies, a three-step inclusion/exclusion criteria were used [14, 23] (Additional file 2). In the first step, children were screened by the principal investigator (YK) using two questions from the ten WHO Screen Questions (Questions 1 and 5) that correlate with motor disability [20]. Next, each one of those who screened positive on the two WHO screening questions underwent further assessments by one of the four attending physicians, i.e. YK, ET, and other two medical doctors, assisted by an observer flow chart, adapted from the ‘Decision Tree’ in SCPE [19]. Finally, all the children were reassessed by either one of the senior pediatricians (ZO or MM), deciding their inclusion or exclusion.

Once the definition of CP and other inclusion criteria were fulfilled, the children were subtyped according to their predominant movement disorders using the classification tree of the SCPE [19], and the subtypes were verified by the senior pediatricians (ZO & MM). The subtypes were (a) bilateral spastic CP; (b) unilateral spastic CP; (c) dyskinetic CP and (d) ataxic CP subtypes. Bilateral spastic CP was further classified into spastic diplegic CP and quadriplegic CP. The severity of gross motor function impairments was classified with the Gross Motor Function Classification System (GMFCS) [6, 24].

The study interviews were conducted in the local language (Tigrigna) after explaining the aim of the study and obtaining informed consent. None of the caregivers of the included children with CP declined consent. All ethical and professional considerations were followed throughout the study to keep the data in strict confidence. The principal investigator (YK) checked on-site for the completeness of the collected data.

### Statistical analyses

Data were entered into the Statistical Package for Social Sciences (SPSS) version 26 for statistical analysis. It was checked twice and cleaned to minimize keying errors. Descriptive statistics were performed as appropriate to characterize the sociodemographic characteristics; antenatal, perinatal, and postnatal risk factors; CP subtypes; GMFCS levels; and the associated comorbidities. Differences in proportions were evaluated using chi-square statistics and logistic regression, both at univariate and multivariate levels. *P*-values < 0.05 were considered statistically significant.

The STROBE checklist for cross-sectional studies [25] was used to appropriately report the study. (Additional file 3)

## Results

**Fig. 1:**
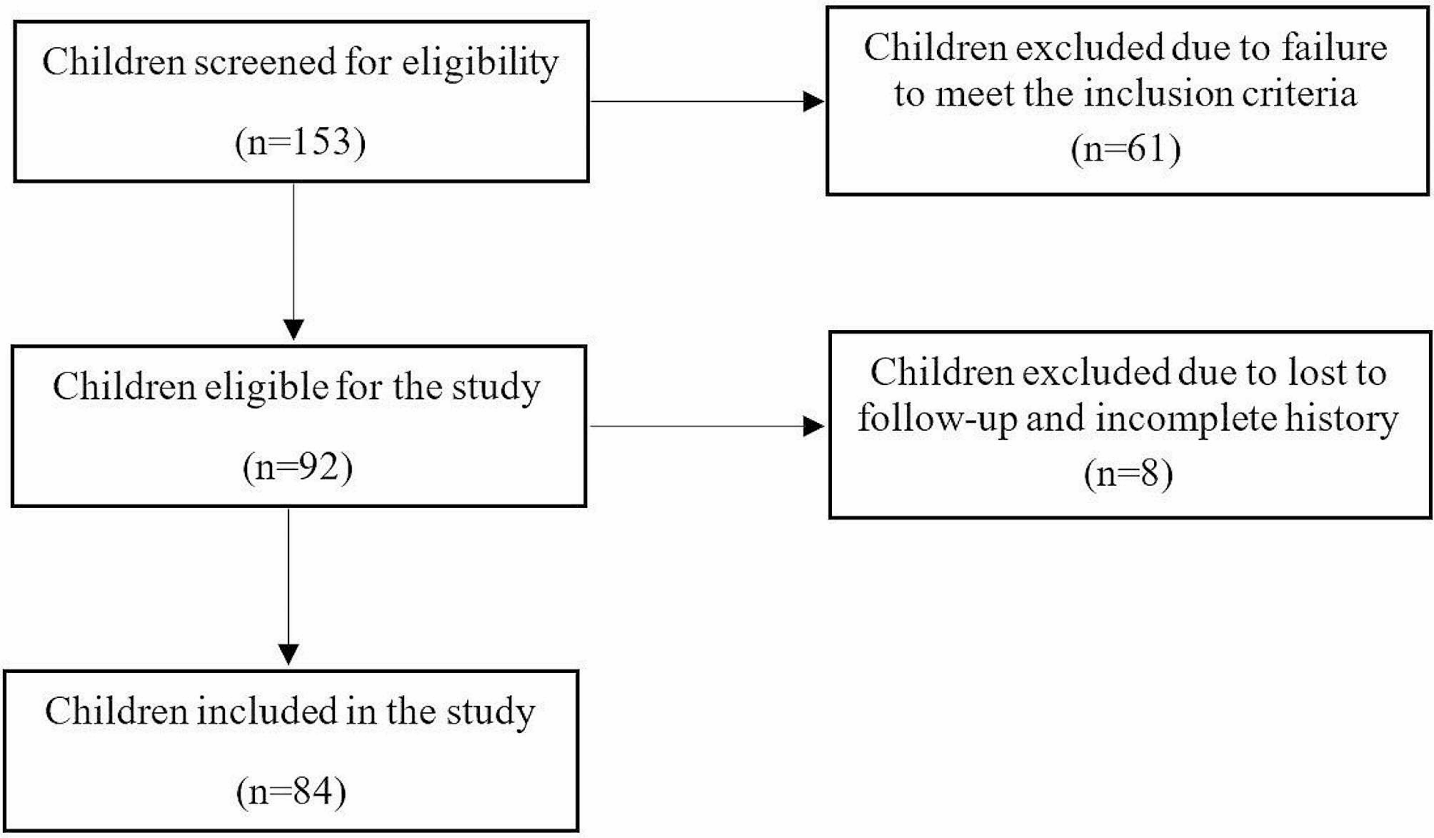
Summary of the study participants’ enrolment

In total, 153 children were screened and 84 were included in the study. Most children were referred from their local hospitals, while some were self-referrals. Sixty-nine children were excluded for several reasons detailed below. Two children had no motor problems; 20 children were out of age limits (17 were less than two years and three were above 12 years); 39 children did not fulfill the criteria or definition of CP; four children’s caregivers were unable to provide the full history; and four discontinued the physiotherapy before completing the assessment. The enrolment flow chart is depicted in Fig. 1.

### Sociodemographic characteristics of the study participants

The median age of the study participants was 5-years [IQR: 3.8], with an equal distribution of males and females. The majority of the caregivers were married, housewives, and had completed some level of formal education (92.9%). In most of the participants’ households, the father’s salary was the main source of income, and mothers were the primary caregivers in 87% with an average of two other children living in the house. The sociodemographic characteristics of the study participants are presented in Table 1.

**Table 1:** Sociodemographic characteristics of children with cerebral palsy visiting Orotta National Referral and Teaching Hospital

Characteristics		*N* (%)
**Age** (median in years [IQR])		5 [3.8]
**Sex**		
	Female	42 (50)
	Male	42 (50)
**Caregiver’s Marital status**		
	In marriage	68 (81)
	Out of marriage	16 (19)
**Caregiver’s education level**		
	None	6 (7.1)
	Preschool to middle school	34 (40.5)
	Secondary and above	44 (52.4)
**Caregiver’s occupation**		
	Housewife	64 (76.2)
	Self employed	9 (10.7)
	Hired	11 (13.1)
**Main source of income**		
	Both parents	13 (15.5)
	Father mainly	41 (48.8)
	Family support	17 (20.2)
	Charity	8 (9.5)
	Self	5 (6.0)
**Children living with CP child** (n)		2

### Clinical subtypes and severity of gross motor comorbidities

Quadriplegic CP was the most common subtype (51.2%) followed by unilateral (hemiplegic) CP (22.6%), and dyskinetic CP (14.3%). Slightly more than two-fifth of the children (43.1%) had severe gross motor impairment GMFCS level IV-V. Table 2 shows the distribution of CP subtypes and GMFCS levels.

**Table 2:** Distribution of cerebral palsy subtypes and Gross Motor Function Classification System levels in children with cerebral palsy visiting Orotta National Referral and Teaching Hospital

CP subtype	*N* (%)
Hemiplegic CP	19 (22.6)
Diplegic CP	5 (6)
Quadriplegic CP	43 (51.2)
Ataxic CP	4 (4.8)
Dyskinetic CP	12 (14.3)
**GMFCS level**	**N (%)**
l	0
ll	19 (22.6)
lll	28 (33.3)
lV	19 (22.9)
V	17 (20.2)

### Antenatal, perinatal, and postnatal risk factors of the study participants

The median mothers’ age was 29 years [IQR: 8]. None of the mothers had the habit of smoking or drinking alcohol. From 13 different antenatal risk factors for CP considered in the study, infection during pregnancy, estrogen/progesterone use, and poor nutrition during pregnancy were the highest recorded possible antenatal risk factors.

To have an insight of the influence of perinatal period on CP development, information on several risk factors was collected. In this study, nine children (11.9%) were delivered at home. Most children (96.4%) were singletons, and out of the two twin deliveries, one child died in the neonatal period. Of the participants, one-tenth were born preterm. The duration of labor was less than 24 h in 92.9%, and PROM was recorded in 10.7% of cases. Out of all the mothers, 38.1% reported difficulty during labor and/or delivery (problems such as prolonged or obstructed labor, PROM, antepartum hemorrhage etc.) Breathing support was given to 35.7% of the children immediately after birth, and 53.3% of cases were admitted to the neonatal intensive care unit (NICU) within 24 h of birth, with an average of 5 days stay. Only one child was born with less than 1 kg. During the perinatal period, almost half of the children (47.6%) had trouble feeding and 14% of the participants had seizures. Out of the whole study participants, 52.4% of the children either did not cry within five minutes and/or needed breathing resuscitation immediately after birth.

Trouble feeding (28.6%), jaundice (6%), and difficulty breathing (10.7%) were found to occur less during the postnatal period than the perinatal period. On the other hand, the occurrence of infection (26.2%) and seizure (20.2%) was higher in the postnatal period. Table 3 shows the commonly reported risk factors in children with CP.

**Table 3:** Common risk factors of children with cerebral palsy visiting Orotta National Referral and Teaching Hospital

Prenatal		Perinatal				Postnatal		
Risk factor	*N* (%)	Risk factor			*N* (%)	Risk factor	*N* (%)	
Mother’s age (Median in years [IQR])	29 [8]	**Place of birth**				Trouble feeding	24 (28.6)	
			Hospital		64 (76.2)	Infection* or fever	22 (26.2)	
Father’s age (Median in years [IQR])	38 [12]		Clinic		9 (11.9)	Seizures	17 (20.2)	
			Home		9 (11.9)	Head trauma	11 (13.1)	
Alcohol intake and smoking habit	0	Single birth			80 (95.2)	Difficulty breathing	9 (10.7)	
		Twin delivery			3 (3.6)	Jaundice	5 (6.0)	
High blood pressure	7 (8.3)	Death of a twin			1 (1.2)			
Diabetes	1 (1.2)	**Gestational age**						
Infection during pregnancy	37 (44.1)		Term		71 (89.9)			
Bleeding during pregnancy	4 (4.8)		> 3 weeks early		8 (10.1)			
Injuries during pregnancy	6 (7.1)	Prolonged rupture of membrane			9 (10.7)			
Poor Nutrition	9 (10.7)							
Thyroid treatment	0	**Duration of labor**						
Estrogen/progesterone use	16 [19]			< 24 h	78 (96.3)			
History of epilepsy	1 (1.2)			> 24 h	3 (3.7)			
Problems conceiving	5 (6.0)	**Delivered by**						
				Midwife	56 (66.7)			
				Doctor	15 (17.9)			
				Traditional birth attendant	5 (6)			
				Family	4 (4.8)			
				Others	3 (3.6)			
		**Mode of delivery**						
				SVD	65 (77.4)			
				Elective C/S	2 (2.4)			
				ER C/S	11 (13.1)			
				Instrumental delivery	4 (4.8)			
		Difficulty in labor			32 (38.1)			
		Baby crying after 5 min of delivery			36 (42.9)			
		Breathing support			30 (35.7)			
		Baby needed help in the first 24 h			37 (44)			
		Duration of NICU stay (Median in days [IQR])			5 [12]			
		Birth weight (Median in Kg [IQR])			2.8 [1.1]			
		**Events in the first 7 days of delivery**						
				Trouble feeding	40 (47.6)			
				Seizure	12 (14.3)			
				Jaundice	12 (14.3)			
				Infection or fever	7 (8.3)			
				Difficulty breathing	26 [31]			

*Infection includes meningitis,* encephalitis*,* and septicemia.*

### Comorbidities associated with the CP

The majority of the study participants (64.3%) had feeding problems, trouble with chewing (44%), and swallowing (39.3%). Of all the CP patients, 47.6% had to be fed by their caregivers, and 40.5% could only eat food with liquid or semisolid consistency, making them unable to eat regular family food.

Only 8.3% of the study participants could express themselves with no problem. The remaining had speech problems with more than half having severe speech disability, communicating only with vocalization and/or crying. On the other hand, a greater percentage of the CP patients (62.6%) had little to no problem understanding when their caregivers spoke to them. School attendance was not common amongst CP patients with only 15.5% attending and most of them performed lower than their peers.

Almost half of the participants (46.4%) had at least one episode of seizure in their lifetime, with most suffering from generalized tonic-clonic seizure. Of those patients, 41% were on anti-epileptic medication. Table 4 shows the distribution of comorbidities associated with the CP.

**Table 4:** Comorbidities in children with cerebral palsy visiting Orotta National Referral and Teaching Hospital

Comorbidity		*N* (%)
**Previous history of seizure**		39 (46.4)
	GTCT seizure	19 (22.6)
	Partial seizure	13 (15.5)
	Complex seizure	4 (4.8)
	Other	3 (3.6)
**Consistency of feeds**		
	Liquids only	3 (3.6)
	Semi-Solids only	31 (36.9)
	No problem	50 (59.5)
**Self-feeding**		
	Skillfully	19 (22.6)
	Unskilled	25 (29.8)
	Must be fed	40 (47.6)
No feeding problems		30 (35.7)
Swallowing problems		33 (39.3)
Chewing problems		37 (44)
Vomiting		4 (4.8)
Drooling		25 (32.1)
**School attendance**		13 (15.5)
	As per age	3 (3.6)
	Lower than peers	10 (11.9)
**School performance**		
	Average	6 (7.1)
	Less than average	6 (7.1)
	Above average	1 (1.2)
**Mode of communication**		
	Nonverbal	36 (42.9)
	Verbal and nonverbal	48 (57.2)

To explore the influence of demographic characteristics on the CP subtype and GMFCS, chi-square and Fischer’s exact test were performed. Among the 11 variables tested (such as gender, caregivers’ educational level…etc), none were found to be associated with the CP subtype. However, two variables, sex and education level, were significantly associated with the GMFCS level. To further determine the direction and magnitude of the influence of those found to be significant in the chi-square test, multivariate logistic regression was conducted. Females were almost three times more likely to have a severe gross motor function GMFCS level IV/V than males (AOR: 2.70; 95% CI: 1.08–6.72; *p*-value = 0.033.) (Table 5).

**Table 5:** Multivariable association of GMFCS level with demographic characteristics

Variable		AOR	95% CI	*p*-value
**Sex**				
	Female	2.70	1.08–6.72	0.033
	Male	*Ref*		
**Educational level**				
	Junior and below	0.78	0.18–1.10	0.078
	Secondary and above	*Ref*		

*Ref: reference category*

## Discussion

This study found spastic CP, dominated by quadriplegic CP, to be the most common CP subtype, a finding consistent with other studies done in LMICs [2, 3, 17, 18, 26, 27]. Additionally, most children had moderate to severe disability, with GMFCS level III through V, similar to other hospital-based cross-sectional studies done in LMICs [2, 3, 28]. On the contrary, less severe disability was dominant in population studies done in Bangladesh [5] and Uganda [29]. The reason that most of the CP patients fell under the category of severe gross motor function can be partly explained by the fact that caregivers usually tend to bring the severely affected children to a higher health facility or due to the fact that caregivers delay in bringing their children to a hospital, by which time the motor function might have worsened. Another reason might also be that the rehabilitative treatment given in LMIC is not in line with most international guidelines. This study also illustrated that females had worse motor functional abilities although a recent literature review found that the statistically significant difference in severity of motor outcomes based on sex is minimal [12]. Hence, a further population-based study is necessary to investigate whether females tend to have worse motor functional disabilities. This study also showed an equal male to female distribution in contrary to other literatures which showed a male predominance [2, 3, 12, 18, 28–30].

Even though none of the antenatal risk factors was found to be significantly associated with the incidence of CP, infection, estrogen/progesterone use, poor maternal nutrition due to severe hyperemesis, and high-blood pressure during pregnancy were prominently recorded in this study. Infections mainly of the genitourinary tract and possibly TORCH infections could also have played a role as they were reported more often than in some other studies [2, 31]. It was also noted that 10.1% of the study participants were born preterm. This finding was comparable to other studies done in other LMICs, such as Nigeria and Benin as opposed to HIC where preterm birth is the main risk factor for CP constituting around 40% of the CP cases [2, 3, 5, 29]. The high number of mothers who recounted difficulty during labor, along with more than half of children who suffered from possible asphyxia points to perinatal factors as the main cause of CP in this setting and other LMICs. Post-neonatal CP in HIC is usually due to head injury followed by infections [32, 33]. In contrast, infections and kernicterus are the major cause of postnatal cases of CP in LMICs. In this study, infection and seizures were reported as the main postnatal risk factors of CP, although seizures can also be a consequence of CP [34, 35].

There were multiple comorbidities associated with CP, epilepsy being the most common one. Seizures occurred in almost half of the children - a finding comparable to a population study done in Uganda, and Nigeria. However, it was higher than that of Benin (18.8%), North India (32%) and Gujranwala- Pakistan (36.2%) [2, 3, 17, 26]. Almost two-thirds of the children in this study had problems with feeding, mostly swallowing and chewing. This number might be underestimated as caregivers may not notice the severity of their child’s feeding problem [36, 37]. Schooling of children with disabilities presents a serious challenge in LMICs. This study showed that 84.5% of the study participants did not attend school inferring how the majority of children with disabilities are not in school. Of those who do attend, almost all of them are in classes lower than their age group. Other LMICs report similar findings, with 70% of the CP cases not attending school in Ethiopia and only 23.7% going to school in Benin [2, 3].

The findings of this study have outlined the following implications: perinatal factors, mostly birth asphyxia, were highly reported. These perinatal factors are usually associated with cortical or basal ganglia injuries and affect children more severely than periventricular lesions [2, 38]. Perinatal risk factors which are the leading risk factors in LMICs are preventable through timely and appropriate interventions. Thus, this study highlights the need for hospital deliveries, better fetal monitoring during labor, and early intervention in the face of any complications; since the few hours from the onset of labor to delivery might define how the entire family lives for the rest of their lives [39]. Mothers also need to be well educated on identifying the early symptoms and signs of infection and jaundice. CP creates a lot of hardships for both the patients and their families due to the motor disabilities and the associated problems such as seizures, and feeding and speech problems. Besides, it is important to note that nutritional rehabilitation could help improving the child’s quality of life and functional ability [36, 40]. These findings, therefore, emphasize the necessity to strengthen the social welfare sector of the nation to greatly improve the living conditions of children with CP.

### Limitations of the study

Although this study used contemporary international systems for the diagnosis of CP and assessment of motor function level to make it standard and comparable with other studies done worldwide, it has the following limitations. The severity of the CP might be overestimated since the study was conducted at Orotta National Referral and Teaching Hospital; a hospital more prone to receive severe cases. The burden and restrictions of COVID-19 have also played a role in decreasing the total number of CP patients visiting the hospital. Another limitation was that much of the information on risk factors and associated comorbidities was based on interviews of the caregivers which could lead to recall bias. Finally, as the study was conducted in a single hospital, caution should be exercised while generalizing the findings. Population-based and longitudinal studies should be conducted for proper representation and to obtain a profound understanding of CP in Eritrea.

## Conclusions

The clinical profile of the CP patients was found to be dominated by spastic sub-type and moderate to severe disability. It was outlined that perinatal risk factors are the most common risk factors of CP. Hence, strengthening maternal and child healthcare systems is recommended to minimize occurrences of the preventable risk factors, and subsequently the burden of permanent disability. It is also recommended to establish standardized and continuously updated CP registers, and conduct a population-based study to determine the prevalence, incidence, and other epidemiological features of CP.

### Electronic supplementary material

Below is the link to the electronic supplementary material.

Supplementary Material 1

Supplementary Material 2

Supplementary Material 3

## Data Availability

The complete dataset used and/or analyzed during the current study are available from the corresponding authors and can be accessed upon a reasonable request.

## Abbreviations

AOR: Adjusted Odds Ratio
APGAR: Activity, Pulse, Grimace, Appearance, Respiratory
CI: Confidence Interval
CP: Cerebral Palsy
HIC: High-income Countries
IQR: Interquartile range
LMIC: Low- and Middle-Income Countries
GMFCS: Gross Motor Functional Classification System
NICU: Neonatal Intensive Care Unit
PROM: Prolonged Rupture of Membrane
SCPE: Surveillance for Cerebral Palsy in Europe
SD: Standard Deviation
SPSS: Statistical Package for Social Sciences
STROBE: The Strengthening the Reporting of Observational Studies in Epidemiology
TORCH: Toxoplasmosis, Other infections, Rubella, Cytomegalovirus, HIV
WHO: World Health Organization
