# Supplementary material for: Clinical profile and associated comorbidities of cerebral palsy in children visiting Orotta National Referral Hospital, Eritrea: a cross-sectional study
Source: BMC Pediatr. 2024 Jul 18;24:458. doi: 10.1186/s12887-024-04938-1 (PMC11256470; doi:10.1186/s12887-024-04938-1)
Supplement: Supplementary file 1 — Supplementary Material 1 [file 12887_2024_4938_MOESM1_ESM.pdf]

Additional file 1: Clinical history questionnaire for the study of '*Clinical profile and associated comorbidities of cerebral palsy in children visiting Orotta National Referral Hospital, Eritrea*'

Cerebral Palsy Study (CPSTUD) ID: ________________

CLINICAL HISTORY QUESTIONNAIRE

1. PERSONAL DETAILS

Identifier	Data	Code
Today's Date *(dd/mm/yyyy)*	__ __ / __ __ / 202 __	**TDATE**
CPSTUD 2022 Number	I G D 2 1 __ __ __ __ __	**CPIGD22**
Patient ID Number	[__] [__] [__] [__] [__] [__] [__]	**PID**
Name of the child	________________________	**NAME**
Name of the residence zone	________________________	**RESID**
Date of birth *(dd/mm/yyyy)*<br>*(If not known, don't estimate. Enter 99/99/9999)*	__ __ / __ __ / __ __ __ __	**DOB**
Age *(in completed years & months)*<br>*(estimate if date of birth is unknown)*	[__] [__] . [__] [__]	**AGE**
Sex *(tick on the appropriate box)*	[M] [F]	**SEX**
Caregiver's Name	________________________	**HEADHSE**
Primary phone number	[__] [__] [__] [__] [__] [__] [__] [__]	**PHCONT1**
Alternative phone number	[__] [__] [__] [__] [__] [__] [__] [__]	**PHCONT2**
Marital status	Married .... 1<br>Single .... 2<br>Widowed .... 3<br>Separated/Divorced .... 4<br>Other *(please specify)* __________ 5	**MARSTAT**
Highest level of education	None .... 1<br>Preschool .... 2<br>Primary (1st – 5th grade) .... 3<br>Junior (6th – 8th grade) .... 4<br>Secondary (9th – 12th grade) .... 5<br>Post-secondary (12th + technical/vocational training) .... 6<br>Tertiary (university) .... 7<br>Unknown 9	**HIGHEDUC**
Major occupation	________________________	**MAJOCCUP**
Family's main source of income	________________________	**MAINCOME**

Family's total monthly income	____________________ ERN	**MONINCOME**
Who will answer questions about the index?	Index's mother................................1<br>Index's father..................................2<br>Index's aunt....................................3<br>Index's uncle...................................4<br>Index's grandmother ....5<br>Index's sibling.................................6<br>Another relative...............................7<br>Other *(please specify)* __________ *8*	**QCHILD**
Who mainly takes care of the Index?	________________________	**INFORM**
How many other children stay at home with this child?	[__] [__]	**CHILDHME**
Name of history taker	__________________________	**HISTNAME**
Date of completion *(dd/mm/yyyy)*	__ __ / __ __ / 202 __	**DOC**

2. PREGNANCY HISTORY (For this child)

**Question**	**Response**	**Code**
Gravidity *{total number of pregnancies before this child (and counting this child)}*	[__] [__]	***CHQ3.01***
Stillbirths *{Note any stillbirths that applied at the time of this child's birth}*	[__] [__]	***CHQ3.02***
Parity *{total number of actual births before this child (and counting this child)}*	[__] [__]	***CHQ3.03***
Spontaneous abortions *{Note any spontaneous abortions that applied at the time of this child's birth}*	[__] [__]	***CHQ3.04***
When the mother was pregnant with this child, did she have:		
Habit of alcohol consumption	Yes..1<br>No *(skip to CHQ3.07)* ....2<br>Unknown......................................99	***CHQ3.05***
Type/name of alcohol	__________________________	***CHQ3.06***
Habit of smoking cigarette or other recreational drugs	Yes..1<br>No *(skip to CHQ3.07)* ....2<br>Unknown......................................99	***CHQ3.07***
Type/name of cigarette or recreational drug	__________________________	***CHQ3.08***
High Blood Pressure (pre-eclampsia)	Yes..1<br>No..2<br>Unknown......................................99	***CHQ3.09***

Bleeding in the 1st trimester	Yes....1<br>No....2<br>Unknown....99	***CHQ3.10***
Bleeding in the 2nd trimester (12th - 28th week of gestation)	Yes....1<br>No....2<br>Unknown....99	***CHQ3.11***
Bleeding in the 3rd trimester	Yes....1<br>No....2<br>Unknown....99	***CHQ3.12***
Diabetes	Yes....1<br>No....2<br>Unknown....99	***CHQ3.13***
Infection or fever in the 1st trimester	Yes....1<br>No....2<br>Unknown....99	***CHQ3.14***
Infection or fever in the 2nd trimester	Yes....1<br>No....2<br>Unknown....99	***CHQ3.15***
Infection or fever in the 3rd trimester	Yes....1<br>No....2<br>Unknown....99	***CHQ3.16***
Injuries resulting from accidents/ violence	Yes....1<br>No....2<br>Unknown....99	***CHQ3.17***
Nutrition	Good....1<br>Moderate....2<br>Poor....3<br>Unknown....99	***CHQ3.18***
Good nutrition is balanced diet which has combination of all nutrients i.e. protein, carbs, fat, vits, min and vegies in a week's meal with at least 3x/day and no history of severe hyperemesis gravidarum.		
Treatment with thyroid hormone	Yes....1<br>No....2<br>Unknown....99	***CHQ3.19***
Treatment with estrogen/ progesterone hormone	Yes....1<br>No....2<br>Unknown....99	***CHQ3.20***
Treatment with anticonvulsants	Yes....1<br>No....2<br>Unknown....99	***CHQ3.21***
Treatment for a psychiatric disorder	Yes....1<br>No....2<br>Unknown....99	***CHQ3.22***
Any other health problems?	Yes *(please specify)* ____________ 1<br>No....2<br>Unknown....99	***CHQ3.23***
** Prompt for signs of UTI. Do not include here problems with veins, moderate vomiting or mild conditions.*		
Has the mother *ever* had a goiter?	Yes....1<br>No....2<br>Unknown....99	***CHQ3.24***

Has the mother *ever* had epilepsy?	Yes ... 1<br>No ... 2<br>Unknown ... 99	***CHQ3.25***
Did the mother have problems conceiving?	None ... 1<br>Yes but no intervention ... 2<br>Yes, and assisted with ovulation induction ... 3<br>Yes, and assisted with Advanced Assisted Reproduction Tech. ... 4<br>Unknown ... 99	***CHQ3.26***
Age of father at child's birth (years)	[__] [__]	***CHQ3.27***
Age of mother at child's birth (years)	[__] [__]	***CHQ3.28***

3. BIRTH HISTORY

**Question**	**Response**	**Code**
Where was the child born?	Home ... 1<br>Hospital ... 2<br>Clinic/Maternity Home ... 3<br>Other *(please specify)* ___________ 4<br>Unknown ... 99	***CHQ4.01***
Was it a single birth?	Single birth ... 1<br>Twins ... 2<br>Triplets or more ... 3<br>Unknown ... 99	***CHQ4.02***
Death of a twin in utero or less than two years?	Yes ... 1<br>No ... 2<br>Unknown ... 99	***CHQ4.03***
Was the baby born at term (between 37 - 42 weeks or at about 9 months)?	Yes ... 1<br>No, ≥ 3 weeks early ... 2<br>No, ≥ 2 weeks late ... 3<br>Unknown ... 99	***CHQ4.04***
Was there prolonged rupture of membranes (>18 hours) before delivery?	Yes ... 1<br>No *(specify hours)* ______________ 2<br>Unknown ... 99	***CHQ4.05***
Color of liquor	Clear ... 1<br>Lightly meconium stained ... 2<br>Thick meconium stained ... 3<br>Unknown ... 99	***CHQ4.06***
How long was the labor?	≤ 24 hours ... 1<br>>24 hours ... 2<br>No labor, C-Section ... 3<br>Unknown ... 99	***CHQ4.07***
Who assisted in delivering the baby?	Trained midwife ... 1<br>Traditional birth attendant ... 2<br>Doctor ... 3<br>Family member ... 4<br>Other *(please specify)* ___________ 5<br>Unknown ... 99	***CHQ4.08***

Was there cord prolapse during labor and/or delivery?	Yes ... 1<br>No ... 2<br>Unknown ... 99	***CHQ4.09***
Was there placenta previa during labor and/or delivery?	Yes ... 1<br>No ... 2<br>Unknown ... 99	***CHQ4.10***
Was there uterine rupture during labor and/or delivery?	Yes ... 1<br>No ... 2<br>Unknown ... 99	***CHQ4.11***
Were there any other difficulties with labor and/or delivery?	Yes *(please specify)* ____________ 1<br>No ... 2<br>Unknown ... 99	***CHQ4.12***
What was the Mode of delivery?	SVD ... 1<br>Elective C/S ... 2<br>ER C/S ... 3<br>Instrumental delivery ... 4<br>Unknown ... 99	***CHQ4.13***
In what position was the baby born?	Head first ... 1<br>Feet or buttocks first ... 2<br>C-Section ... 3<br>Unknown ... 99	***CHQ4.14***
What was the APGAR score of the baby?	At 1st minute<br>≤ 5 ... 1<br>> 5 ... 2<br>Unknown ... 99<br>At 5th minute<br>≤ 5 ... 1<br>> 5 ... 2<br>Unknown ... 99	***CHQ4.15***
Did the baby cry immediately after birth?	Yes ... 1<br>No, but in < 5 min ... 2<br>No, after > 5 min ... 3<br>Unknown ... 99	***CHQ4.16***
What was the color of the baby at birth?	Normal ... 1<br>Blue ... 2<br>Pale/white ... 3<br>Unknown ... 99	***CHQ4.17***
Did the birth attendant have to do anything to the baby to make her/him breath?	Yes ... 1<br>No ... 2<br>Unknown ... 99	***CHQ4.18***
If yes, describe **why** and **how** breathing was assisted:<br>____		
Was the baby taken away from the mother immediately after birth?	Yes ... 1<br>No ... 2<br>Unknown ... 99	***CHQ4.19***
If yes, describe the circumstances:<br>____		

If the baby was kept in a hospital, for how many days was he/she kept there? *(Enter 00 if child was not admitted to hospital)*	[__] [__]	***CHQ4.20***
What was the birth weight in kilograms?	[__] [__]	***CHQ4.21***
How big was the baby at birth?	About the size of most babies .... 1<br>Smaller than most babies................. 2<br>Bigger than most babies .... 3<br>Unknown 99	***CHQ4.22***
Did the child have any additional birth anomalies?	Yes *(please specify)*____________ 1<br>No .... 2<br>Unknown 99	***CHQ4.23***

4. PERINATAL HISTORY

(**Perinatal period:** from 28th weeks gestation to the first seven days after birth)

Question	Response	Code
Did the child have any of the below conditions from birth to the first seven days after birth?		
Seizure	Yes.. 1<br>No .... 2<br>Unknown 99	***CHQ4.24***
Infection (fever)	Yes.. 1<br>No .... 2<br>Unknown 99	***CHQ4.25***
Trouble feeding	Yes.. 1<br>No .... 2<br>Unknown 99	***CHQ4.26***
Jaundice	Yes.. 1<br>No .... 2<br>Unknown 99	***CHQ4.27***
Difficulty breathing	Yes.. 1<br>No .... 2<br>Unknown 99	***CHQ4.28***

5. POST-NATAL HISTORY

Question	Response	Code
Did the child have any of the below conditions from the first seven days after birth up to one year of age?		
Seizure	Yes.. 1<br>No .... 2<br>Unknown 99	***CHQ5.01***
Infection (fever)	Yes.. 1<br>No .... 2<br>Unknown 99	***CHQ5.02***
Trouble feeding	Yes.. 1<br>No .... 2<br>Unknown 99	***CHQ5.03***

Jaundice	Yes........1<br>No........2<br>Unknown........99	***CHQ5.04***
Difficulty breathing	Yes........1<br>No........2<br>Unknown........99	***CHQ5.05***
Trauma to head (E.g. MVA, near drowning, non-accidental etc.)	Yes........1<br>No........2<br>Unknown........99	***CHQ5.06***

6. EPILEPSY HISTORY

Question	Response	Code
Has the child ever suffered from seizure or epilepsy?	Yes (verbal account) ____________ 1<br>No *(skip to section 7)* ....2<br>Unknown........99	***CHQ6.01***
Seizure will be defined as single event of brief loss of consciousness with or without motor activities and may be due to a transient abnormality that will not recur (e.g., Hypoglycemia).<br>*Epilepsy: was defined as a condition of unprovoked recurrent seizures. The classification of epilepsy in this study was based on seizure description alone, according to the international classification of Epileptic Seizures of 1981.*		
Type of seizure disorder	Generalized........1<br>Partial........2<br>Complex partial........3<br>Unknown........99	***CHQ6.02***
If child has seizure, is he/ she on medication for seizures?	Yes........1<br>No........2<br>Unknown........99	***CHQ6.03***

7. FAMILY HISTORY

Question	Response	Code
Are the parents of the child related to each other by blood?	No........1<br>Yes, as uncle or niece........2<br>Yes, as first cousins........3<br>Yes, as second cousins........4<br>Yes, as distant cousins........5<br>Unknown........99	***CHQ7.01***
Is there any other member of the family with a similar condition?	No........1<br>Yes *(specify who)* ____________ 2<br>Unknown........99	***CHQ7.02***
Is the menstrual character of the mother regular?	Regular........1<br>Irregular........2<br>Unknown........99	***CHQ7.03***
Normal variances include a cycle length between 21 and 35 days, bleeding for up to 7 days, as well as mild to moderate cramping, often relieved with over- the-counter medications.		

8. NUTRITIONAL HISTORY

Question	Response	Code
Was the child breast-fed and for how long?	Never .... 1<br>Yes, ≤ 6 months .... 2<br>Yes, ≤ 12 months .... 3<br>Yes, ≤ 24 months .... 4<br>Yes, > 24 months .... 5<br>Still breast feeding........ 77<br>Unknown 99	***CHQ8.01***
When did the child first start liquid/semisolid feeds other than breast milk?	Never .... 1<br>Yes, ≤ 6 months .... 2<br>Yes, ≤ 12 months .... 3<br>Yes, ≤ 24 months .... 4<br>Yes, > 24 months .... 5<br>Not yet .... 77<br>Unknown 99	***CHQ8.02***
At what age in months was solid food introduced?	Never .... 1<br>Yes, ≤ 6 months .... 2<br>Yes, ≤ 12 months .... 3<br>Yes, ≤ 24 months .... 4<br>Yes, > 24 months .... 5<br>Not yet .... 77<br>Unknown 99	***CHQ8.03***
What are the three main types of foods that child is fed on?<br>*Multiple responses are possible*	Legumes .... 1<br>Bread/potato/rice .... 2<br>Milk .... 3<br>Green vegetables .... 4<br>Fish .... 5<br>DMK........ 6<br>Tea .... 7<br>Tihni/cereals .... 8<br>Meat........ 9<br>Porridge .... 10<br>Other *(please specify)* _________ 11<br>Unknown 99	***CHQ8.04***
How often is child fed with above (CHQ8.04) food within a 24-hour period?	Once .... 1<br>Twice........ 2<br>Thrice .... 3<br>Four times........ 4<br>More than 4X/day........ 5<br>Unknown 99	***CHQ8.05***
Note: Write your remark based on CHQ (8.04 and 8.05): Good = 1 Moderate = 2 Poor = 3		
What is the consistency of the feeds?	Liquids........ 1<br>Semi-solids........ 2<br>Solids........ 3<br>Both solids and liquids 4	***CHQ8.06***

Can the child feed himself or herself?<br>*(assess in accordance to local cultural norms)*	Yes, skillfully with spoon/fork or fingers ... 1<br>Yes, but unskilled (i.e. like a baby) ... 2<br>No, must be fed ... 3<br>Unknown ... 99	***CHQ8.07***
Does child have any feeding problems?	Yes ... 1<br>No *(skip to CHQ8.10)* ... 2<br>Unknown ... 99	***CHQ8.08***
If child has a feeding problem, which one of these is the most dominant one?	Swallowing difficulties ... 1<br>Chewing difficulties ... 2<br>Regurgitation of feeds ... 3<br>Vomiting of feeds ... 4<br>Tongue thrust ... 5<br>Drooling saliva ... 6<br>Tonic bite ... 7<br>Unknown ... 99	***CHQ8.09***
What is the weight of the child in Kg?	____________	***CHQ8.10***
What is the height of the child in cm?	____________	***CHQ8.11***
What is the MUAC of the child?	____________	***CHQ8.12***
Weight for height of the child	< -3SD ... 1<br>≥ -3SD and < -2SD ... 2<br>≥ -2SD ... 3	***CHQ8.13***
Weight for age of the child	< -3SD ... 1<br>≥ -3SD and < -2SD ... 2<br>≥ -2SD ... 3	***CHQ8.14***
Height for age of the child	< -3SD ... 1<br>≥ -3SD and < -2SD ... 2<br>≥ -2SD ... 3	***CHQ8.15***
What is the BMI of the child?	< 5 percentile ... 1<br>≥ 5 and < 85 percentile ... 2<br>≥ 85 and < 95 percentile ... 3<br>≥ 95 percentile ... 4	***CHQ8.16***

9. DEVELOPMENTAL HISTORY

**Question**	**Response**	**Code**
At what age did child attain the first social smile?	By 6 weeks ... 1<br>By 4 months ... 2<br>By 6 months ... 3<br>By 8 months ... 4<br>By 1 year ... 5<br>Not yet ... 77<br>Unknown ... 99	***CHQ9.01***

At what age did child sit without support?	By 9 months .... 1<br>By 12 months .... 2<br>By 18 months .... 3<br>By 24 months .... 4<br>By 36 months .... 5<br>Not yet .... 77<br>Unknown 99	***CHQ9.02***
At what age did the child walk without help or holding on?	By 18 months .... 1<br>By 2 years .... 2<br>After 2 years .... 3<br>Not yet .... 77<br>Unknown 99	***CHQ9.03***
At what age did child start to finger feed himself or herself?	By 7 months .... 1<br>By 1 years .... 2<br>By 18 months .... 3<br>By 2 years .... 4<br>After 2 years .... 5<br>Not yet .... 77<br>Unknown 99	***CHQ9.04***
At what age did child start speaking two word sentences?	By 24 months .... 1<br>By 30 months .... 2<br>After 30 months .... 3<br>After 36 months .... 4<br>Not yet .... 77<br>Unknown 99	***CHQ9.05***
What is the grade of the child if s/he goes to school?	As per age .... 1<br>Lower than peers .... 2<br>Higher than peers .... 3<br>Unknown 99	***CHQ9.06***
What is the school performance of the child?	Average .... 1<br>Less than average .... 2<br>Greater than average .... 3<br>Unknown 99	***CHQ9.07***

10. SKIN

**Question**	**Response**	**Code**
Does the child have any pressure sores?	Yes .... 1<br>No 2	***CHQ10.01***

11. SPEECH AND LANGUAGE FUNCTIONS

**Question**	**Response**	**Code**
Child's dominant mode of communication	None .... 1<br>Nonverbal .... 2<br>Verbal .... 3<br>Verbal and Nonverbal 4	***CHQ11.01***

Child's verbal expression	None/only crying............................1<br>Vocalization ....2<br>Words ....3<br>Phrases..4<br>Sentences.......................................5	***CHQ11.02***
Child's verbal comprehension	None ....1<br>Words ....2<br>Phrases..3<br>Simple sentences ....4<br>Complex sentences.........................5	***CHQ11.03***
Child's nonverbal expression	None ....1<br>Poor ....2<br>Gestures..3<br>Signs...4	***CHQ11.04***
Child's nonverbal comprehension	None ....1<br>Poor ....2<br>Gestures..3<br>Facial expressions ....4<br>Signs...5	***CHQ11.05***
Child's Intelligibility of speech	None ....1<br>Poor ....2<br>Fair ....3<br>Good...4	***CHQ11.06***

12. OBSERVATION OF FUNCTION

Complete for all children. Instructions: Observe the child carry out the tasks listed below:

1. Observe the child walking at least 5 steps into room. Watch carefully, looking for limp, asymmetry of gait, toe walking, ataxia, involuntary movement, and atrophy or contracture.
2. Welcome the child and observe the response: Does he or she hear, make an appropriate social response, smile, act shy, or speak?
3. Elicit speech by asking the child questions such as: "What did you pick up? "What is that?" (point to a raisin, chair etc.) "What is this called?" (point to nose, ear, tooth etc.) "What is your name?" Watch for problems in hearing, speech and comprehension.
4. Ask the child to point to body parts (eyes, mouth etc.) Observe for problems in hearing and comprehension.

**Question**	**Response**	**Code**
Rate the child in the following areas after observing the above tasks:		
Comprehension	Good...1<br>Fair ....2<br>Poor ....3<br>Uncertain 99	***CHQ12.01***
Hearing	Good...1<br>Fair ....2<br>Poor ....3<br>Uncertain 99	***CHQ12.02***

Vision	Good .... 1<br>Fair .... 2<br>Poor .... 3<br>Uncertain 99	***CHQ12.03***
Speech (motor)	Good .... 1<br>Fair .... 2<br>Poor .... 3<br>Uncertain 99	***CHQ12.04***
Speech (language)	Good .... 1<br>Fair .... 2<br>Poor .... 3<br>Uncertain 99	***CHQ12.05***

What cerebral palsy subtype does the child has, based on the interview with the informant and this brief observation?	Spastic hemiplegia/paresis .... 1<br>Spastic diplegia/paresis .... 2<br>Spastic quadriplegia/paresis .... 3<br>Dyskinetic................ 4<br>Ataxic 5	***CHQ12.06***

**Name of the data collector**	
**Signature**<br>*(Sign only after checking that the form is complete)*	
