# Supplementary material for: Clinical profile and associated comorbidities of cerebral palsy in children visiting Orotta National Referral Hospital, Eritrea: a cross-sectional study
Source: BMC Pediatr. 2024 Jul 18;24:458. doi: 10.1186/s12887-024-04938-1 (PMC11256470; doi:10.1186/s12887-024-04938-1)
Supplement: Supplementary file 2 — Supplementary Material 2 [file 12887_2024_4938_MOESM2_ESM.pdf]

Additional file 2: Diagnostic, inclusion and exclusion criteria

STEP 1: Two questions from the WHO Ten Screening Questions (Questions 1 and 5)

1. Compared with other children, did the child have any serious delay in sitting, standing or walking? (ምስ ካልኦት ቆልዑ ብምውድዳር፡ እዚ ቆሎዓ ኣብ ኮፍ ፡ ደው ወይ ምኻድ ኣዝዩ ደንጕዩ’ዶ ነይሩ፧)
2. Does the child have difficulty in walking or moving his/her arms or does he/she have weakness and/or stiffness in the arms or legs? (እዚ ቆልዓ ኣብ ምኻድ ወይ ምንቅስቃስ ናይ መሓውር ጸገም፡ ድኻም ወይ ምድራቅ ኣለዎ’ዶ፧)

STEP 2: Algorithm from SCPE for inclusion or exclusion of cases

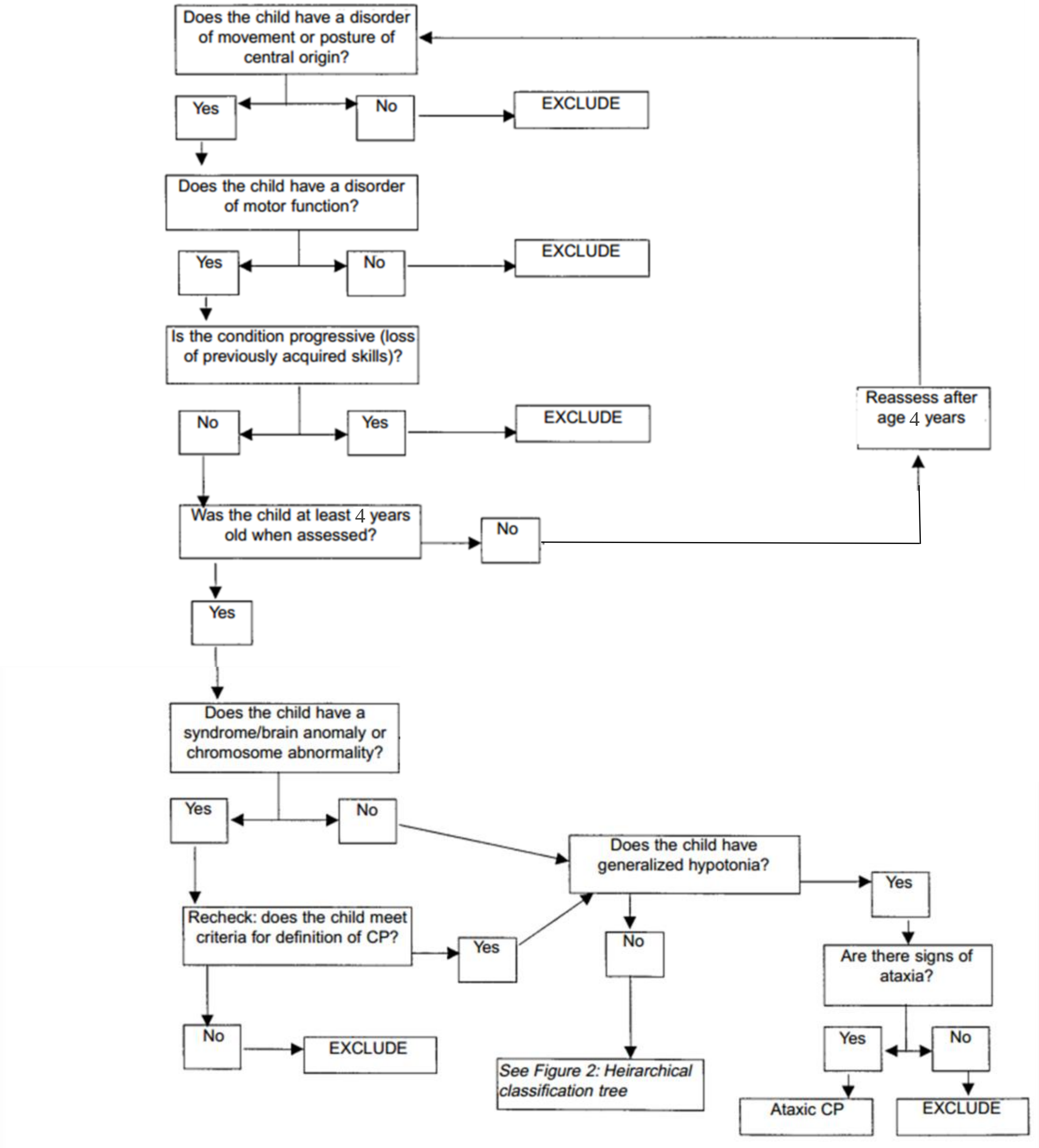

Figure 1: Decision tree for inclusion/ exclusion of cases of cerebral palsy on SCPE register (Cans, 2000).

Assessed by:___________________________ Signature________________ Date: ___________

STEP 3: Final decision from senior Pediatrician to include or exclude a case

Pediatrician's name:______________________________Signature__________ Date:_________

Algorithm from SCPE to classify Cerebral Palsy cases into clinical subtypes

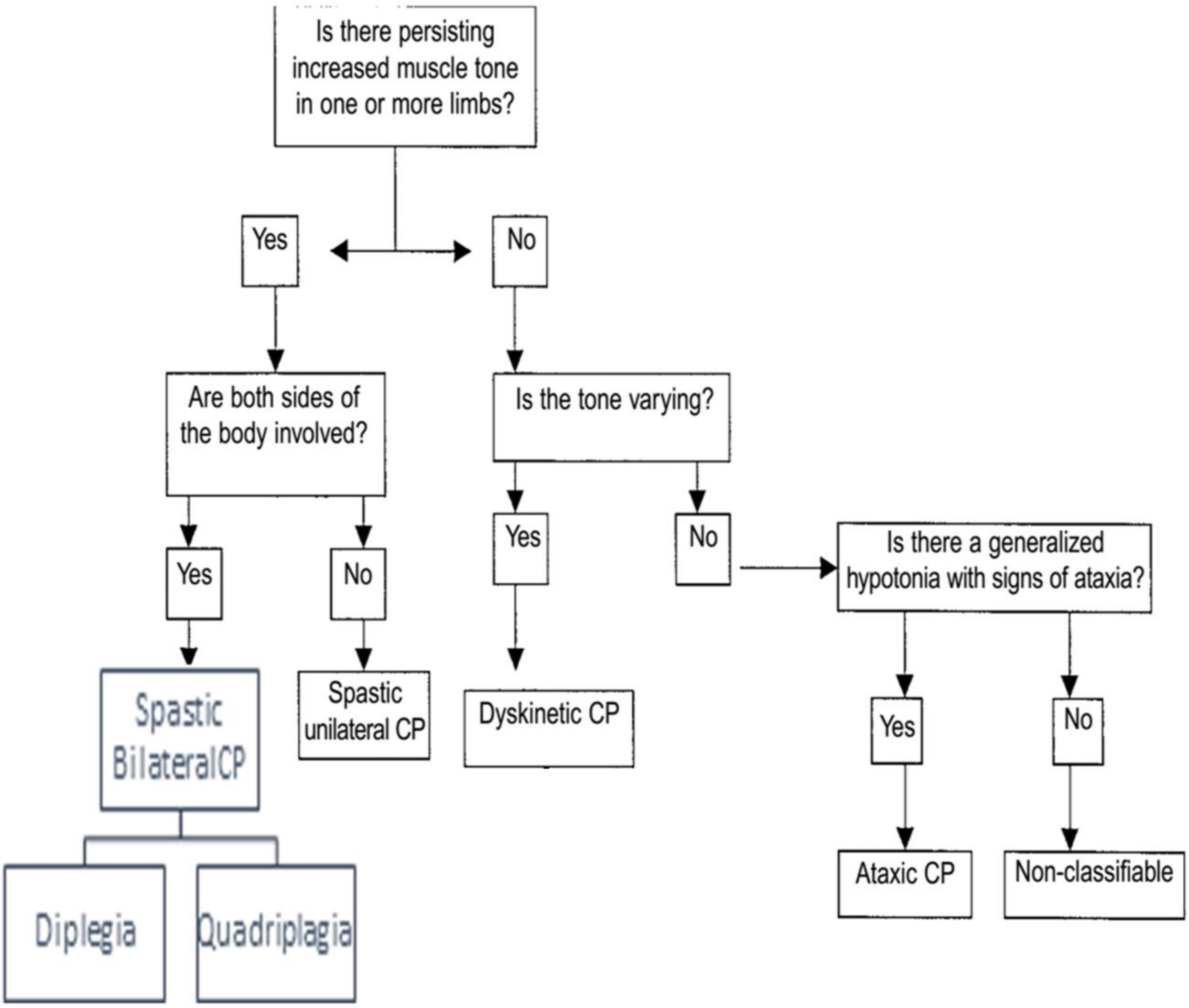

Figure 2: Hierarchical classification tree of cerebral palsy sub-types (Cans 2000; Van Toorn et al., 2007).
